# Route of Infection Determines the Impact of Type I Interferons on Innate Immunity to *Listeria monocytogenes*

**DOI:** 10.1371/journal.pone.0065007

**Published:** 2013-06-19

**Authors:** Elisabeth Kernbauer, Verena Maier, Isabella Rauch, Mathias Müller, Thomas Decker

**Affiliations:** 1 Max F. Perutz Laboratories, University of Vienna, Vienna, Austria; 2 Institute of Animal Breeding and Genetics and Biomodels Austria, University of Veterinary Medicine Vienna, Vienna, Austria; University of Iowa Carver College of Medicine, United States of America

## Abstract

*Listeria monocytogenes* is a food-borne pathogen which causes mild to life threatening disease in humans. Ingestion of contaminated food delivers the pathogen to the gastrointestinal tract, where it crosses the epithelial barrier and spreads to internal organs. Type I interferons (IFN-I) are produced during infection and decrease host resistance after systemic delivery of *L. monocytogenes*. Here we show that mice benefit from IFN-I production following infection with *L. monocytogenes* via the gastrointestinal route. Intragastric infection lead to increased lethality of IFN-I receptor chain 1-deficient (Ifnar1−/−) animals and to higher bacterial numbers in liver and spleen. Compared to infection from the peritoneum, bacteria infecting via the intestinal tract localized more often to periportal and pericentral regions of the liver and less frequently to the margins of liver lobes. Vigorous replication of intestine-borne *L. monocytogenes* in the livers of Ifnar1−/− mice 48 h post infection was accompanied by the formation of large inflammatory infiltrates in this organ and massive death of surrounding hepatocytes. This was not observed in Ifnar1−/− mice after intraperitoneal infection. The inflammatory response to infection is shaped by alterations in splenic cytokine production, particularly IFNγ, which differs after intragastric versus intraperitoneal infection. Taken together, our data suggest that the adverse or beneficial role of a cytokine may vary with the route of infection and that IFN-I are not harmful when infection with *L. monocytogenes* occurs via the natural route.

## Introduction

*Listeria monocytogenes* (Lm) is a food-borne pathogen causing potentially life threatening disease in immunocompromised humans [1]. Lm's ability to traverse epithelial barriers including the intestinal epithelium, the placental and the blood brain barrier is a prerequisite for systemic dissemination and its sequels such as sepsis, fetal abortion and encephalitis. The natural route of infection is via ingestion of contaminated food and although the course of infection is in most cases very mild, manifested disease has one of the highest mortality rates among food-borne diseases. Lm uses a set of virulence factors to successfully invade hosts. These include internalins that allow attaching to, and subsequently invade various different cell types. Internalins (Inl) A and B are instrumental for the invasion of epithelial cells and hepatocytes [2].

Type I interferons (IFN-I) are cytokines essential for the establishment of innate antiviral immunity. Their role in inflammatory disease or bacterial infections varies between protective or detrimental, depending on the pathogen and conditions of infection [3], [4]. Mice lacking the IFN-I receptor show increased resistance to intraperitoneal or intravenous infection with Lm compared to wildtype (Wt) mice [5], [6], [7]. Therefore, IFN-I are thought to decrease the ability of mice to combat infection. The harmful effects of IFN-I have been assigned to different aspects of the immune response. The death of effector cells such as T-cells and macrophages increases with IFN-I signalling during Lm infection [7], [8]. As a consequence of the increased uptake of apoptotic cells interleukin 10 (IL10) is produced which hampers protective immune responses [9]. In line with this, IL10−/− mice are more resistant to Lm infection than Wt mice [10]. In addition, IFN-I may also interfere with IFNγ dependent macrophage activation by decreasing cell surface expression of the IFNγ receptor [11].

The data describing the adverse effect of IFN-I on the course of Lm infection stem from systemic infection models, mostly intravenous or intraperitoneal infection. Immune reactions to gastrointestinal Lm infections, the natural route of infection, have not been studied intensively due to the limitations of the murine system, namely low infection efficiency and incompatibility of cell surface molecules required for invasion. Specifically, murine E-cadherin on epithelial surfaces interacts poorly with the bacterial Internalin A (InlA), an invasion protein located at the surface of Listeria [12], [13].

Here we show for the first time how IFN-I affects infection with Lm via the natural route. Our experiments were carried out with a mouse-adapted Lm strain, which allows robust and efficient infection of mice via intragastric gavage. We clearly demonstrate that the route of infection matters for the impact of IFN-I on innate resistance, as mice benefit from IFN-I after intragastric infection. Differences in splenic cytokine production, inflammatory cell recruitment and hepatotoxicity are suggested to underlie the infection route-dependent impact of IFN-I on the outcome of infection with Lm.

## Materials and Methods

### Mice, bacteria

C57BL/6N (Wt) and Ifnar1−/− (B6.129P2-IfnaR1^tm1^ [14]) mice were housed under SPF conditions. Ifnar1−/− mice used in this study were obtained after more than ten backcrosses to pure C57BL/6N. Animal experiments were discussed and approved by the University of Veterinary Medicine Vienna institutional ethics committee and carried out in accordance with protocols approved by the Austrian law (BMWF-68.205/0204-C/GT/2007; BMWF-68.205/0210-II/10b/2009, BMWF-68.205/0243-II/3b/2011). A scoring-system for assessment of distress of the animals used was established before infection experiments were started. On the basis of these guidelines general condition and behavior of the animals was controlled by well-educated and trained staff (participants of FELASA B training courses). Depending on the progress of the disease, animals were monitored every 3–4 hours during the „day-phase“ (7:00 am to 7:00 pm). In order not to disturb the circadian rhythm of the animals, there was no monitoring after 7:00 pm. Humane endpoint by cervical dislocation was conducted if death of the animals during the following hours was to be expected. Animal husbandry and experimentation was performed under the national laws (Federal Ministry for Science and Research, Vienna, Austria) and ethics committees of the Medical University of Vienna and the University of Veterinary Medicine Vienna and according to the guidelines of FELASA which match that of ARRIVE.

As a prerequisite for constructing the mouse-adapted *Listeria monocytogenes* LO28InlA^S192N/Y369S^ strain we prepared an InlA knockout in LO28wt using the pMAD vector [15] and the following primers for amplification of the upstream and downstream region of the InlA gene from genomic LO28 DNA: InlA_A 5′ CAT GGT CGA CGG CAG TCC GCG ATT TAA TGG AAG T 3′, InlA_B 5′ CAT GGG ATC CCC TAA TCT ATC CGC CTG AAG CGT TGT 3′ InlA_C 5′ CAT GGG ATC CGG GAA TTC AGC CAG CAC AAC AAG T 3′ and InlA_D 5′ CTG CCA TGG AGG TTT AGG TGC AGT TAT CCG CGT 3′. For genomic integration we used the protocol described in [16]. We transformed the LO28InlA knockout strain with the pAUL-A InlA^S192N/Y369S^ -InlB construct kindly provided by WD Schubert (Molecular Host-Pathogen Interactions, Division of Structural Biology, Helmholtz Centre for Infection Research, Braunschweig, Germany [17]) and obtained genomic integration as described in [18]. Here the abbreviation Lm for experiments always refers to LO28InlA*.

### Infection of mice, determination of bacterial organ loads

Bacteria were prepared for infection as described previously [19]. For infection, Lm LO28InlA* were washed, diluted to the respective concentration with PBS (Sigma) and injected intraperitoneally (i.p.) or intravenously (i.v.) into 8- to 10-week-old, sex and age matched C57BL/6N (Wt) and Ifnar1−/− (B6.129P2-IfnaR1^tm1^ [14]) mice. For intragastric infection mice were infected as described [20]. Briefly, mice were starved over night and orally gavaged with CaCO_3_ in 200 µl PBS (50 mg/ml) succeeded by the respective dose of Lm in 200 µl of PBS. The infectious dose was controlled by plating serial dilutions on Oxford agar plates. The survival of mice was monitored for 10 days and data were displayed as Kaplan-Meier plots. For determination of bacterial loads (colony forming units, CFU) of livers, spleens, mesenteric lymph nodes (MLN) and Peyer's patches (PP), mice were sacrificed at the indicated time points, organs isolated and homogenized in PBS. Serial dilutions of the homogenates were plated on BHI plates and incubated at 37°C for 24 h.

### RNA isolation, cDNA synthesis and RT-qPCR

Organs of respective animals were isolated and stored at −80°C until further use. For RNA preparation organs were homogenized in RA1 buffer of the NucleoSpin II RNA isolation kit (Macherey and Nagel) and processed according to protocol. cDNA was prepared as described [21]. The RT-qPCRs were run on an Eppendorf cycler. mRNAs were normalized to the GAPDH housekeeping control and values obtained from infected Wt mice set to 1. Data from Ifnar1−/− mice were obtained and the ratio to the value obtained to respective samples from the Wt controls was calculated. Values in the figures for Ifnar1−/−mice thus represent the relative induction compared to the value obtained at the same time point from Wt animals. Sequences of primers are listed in table I.

**Table 1:** Primer sequences used for qPCR cytokine-induced genes.

IL6	for	TAG TCC TTC CTA CCC CAA TTT CC
	rev	TTG GTC CTT AGC CAC TCC TTC
IL10	for	GGT TGC CAA GCC TTA TCG GA
	rev	ACC TGC TCC ACT GCC TTG CT
Mx2	for	CCA GTT CCT CTC AGT CCC AAG ATT
	rev	TAC TGG ATG ATC AAG GGA ACG TGG
MCP1	for	CTT CTG GGC CTG CTG TTC A
	rev	CCA GCC TAC TCA TTG GGA TCA
GAPDH	for	CAT GGC CTT CCG TGT TCC TA
	rev	GCG GCA CGT CAG ATC CA
IFNβ	for	TCA GAA TGA GTG GTG GTT GC
	rev	GAC CTT TCA AAT GCA GTA GAT TCA
TNFα	for	CAA AAT TCG AGT GAC AAG CCT G
	rev	GAG ATC CAT GCC GTT GGC
IFNγ	for	ATG AAC GCT ACA CAC TGC ATC
	rev	CCA TCC TTT TGC CAG TTC CTC

### Analysis of IFNγ and alanine aminotransferase levels (ALT) in mouse serum

For cytokine analysis mice were bled via the retro-orbital sinuses and serum was prepared and stored at −80°C. IFNγ was measured using the FlowCytomix kit (eBioscience) in 25 µl of serum. ALT concentrations were measured in mouse serum using a COBASc11 analyzer (Roche).

### Cell culture

Bone marrow derived cells were isolated and grown as described [19]. Briefly, bone marrow was isolated from femurs of 6–8 week old mice. For differentiation of bone marrow derived macrophages, cells were grown in DMEM (Gibco, Invitrogen) in the presence of 10% FCS (Gibco, Invitrogen) and L-cell derived CSF-1 as described [19]. The cultures contained >99% F4/80+ cells. Dendritic cells (mDC) were obtained by culture of mouse bone marrow cells in DMEM (Gibco, Invitrogen), supplemented with 10% FCS (Gibco, Invitrogen) and X-6310 derived GM-CSF as described [19]. mDC cultures contained virtually no F4/80+ cells and the purity of CD11c+/CD11b+ cells was between 60 and 70%. Cells were infected with Lm at a MOI of 10 for 1 h, extensively washed and resuspended in PBS for injection into mice.

### Histology

Mouse organs were fixed with 4% paraformaldehyde over night, paraffin embedded and 3 µm sections were prepared using a microtome. Hematoxyline and eosin staining (H&E) were performed using standard protocols. For TdT-mediated dUTP nick end labelling (TUNEL), sections of liver and spleens were stained as described before [22]. For Gr-1 staining liver sections were blocked for endogenous peroxidase activity in 50% methanol with 3% H_2_O_2_ and boiled for 30 min in 10 mM sodium citrate antigen unmasking solution. After cooling down for 30 min, the samples were blocked with 5% normal goat serum to reduce background staining. Primary Gr-1 antibody (BD Pharmingen) was applied overnight at 4°C and before adding AEC+ high sensitivity chromogen substrate (Dako) the sections were incubated with biotinylated rabbit anti-rat IgG (1∶250 in PBS) for 30 min at RT following incubation with ABC reagent (Vector) for 30 min at RT. To stain Lm in infected tissue, sections were blocked for endogenous peroxidase activity in 50% methanol with 3% H_2_O_2_ and incubated with 500 µg/ml pronase (Roche) for 10 min at 37°C, washed with PBS containing 0,05% Tween (PBS-T) and blocked with 5% normal goat serum for 30 min at RT. Next, primary Listeria antibody (1∶100) (Abcam) was applied for 1 h at RT, washed with PBS-T following incubation with HRP rabbit/mouse polymer (Dako) for 30 min at RT. After washing, Lm was visualized by AEC+ high sensitivity chromogen substrate and the cells counterstained with haematoxylin.

### Flow cytometry

For isolation of non-parenchymal cells, mice were sacrificed and the liver was perfused immediately with liver perfusion medium (Gibco, Invitrogen) via the portal vein for 3 min at a speed of 8 ml/min followed by liver digest medium (Gibco, Invitrogen) for 5 min. The liver was removed, minced with scissors in 15 ml ice-cold DMEM+10%FCS+penicillin/streptomycin and filtered through a 70 µm cell strainer. To collect the non-parenchymal cell-enriched supernatant the suspension was centrifuged for 5 min at 50 g. Next, non-parenchymal cells were harvested by centrifugation for 5 min at 300 g and washed. After red blood cell lysis the cells were blocked with anti-mouse CD16/32 and stained for Ly6G (BD-Pharmingen), CD11b, Ly6C, F4/80, CD3, CD45 and with eFluor fixable viability dye (eBioscience). Flow cytometry was performed using BD FACSAria.

### Statistical analysis

Bacterial loads of organs were compared using the Mann-Whitney test and indicate the median. mRNA expression data, serum protein levels and histological data were analysed with the Students t Test and indicate the mean and SEM. For both the GraphPad Prism (Graphpad) was used. Asterisks describe statistical significance as follows: *p≤0,05; **p≤0,01; ***p≤0,001

## Results

### IFN-I signalling increases resistance to *Listeria monocytogenes* contracted via the intragastric route

To assess whether the impact of IFN-I on innate immunity to Lm is determined by the infection route, we administered Lm to Wt and Ifnar1−/− mice both by intragastric gavage (i.g.) and intraperitoneal injection (i.p.). These experiments were performed with a mouse-adapted, mutated LO28 strain of Lm, LO28InlA* (see materials and methods). This strain expresses an InlA mutant with increased affinity to murine E-cadherin [17]. Compared to LO28 Wt, intragastric administration of LO28InlA* produced a roughly 5-fold higher bacterial burden in the liver and about 3-fold higher burden in spleen 48 h after infection (Fig. S1). This difference is similar to or larger than that reported for the mouse-adapted EGD strain [17], [23]. By contrast, infection rates were virtually indistinguishable 48 h after i.p. infection (Fig. S1).

**Figure 1:**
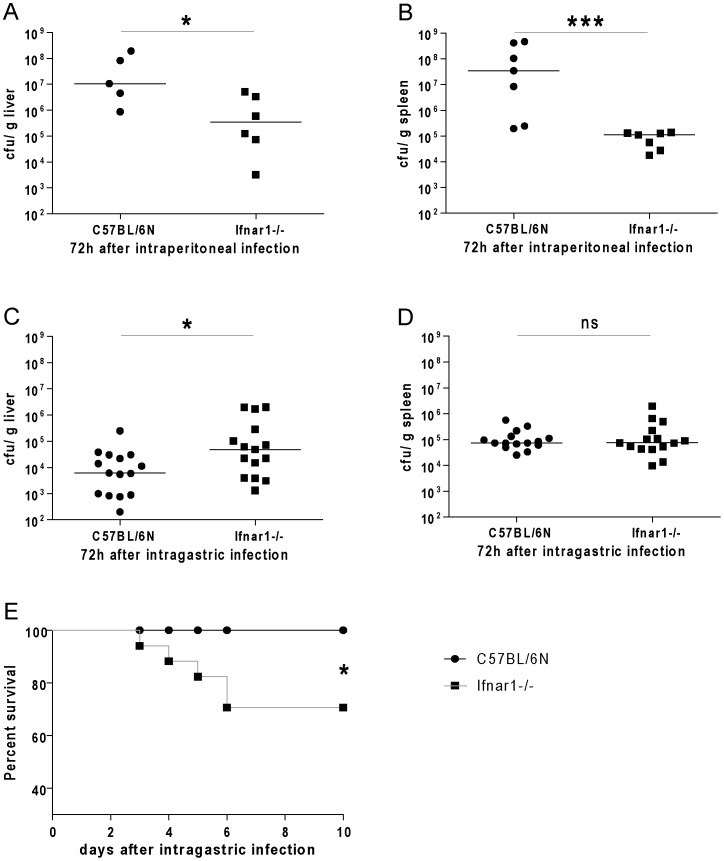
IFN-I increase host resistance after intragastric infection with *Listeria monocytogenes*. C57BL/6N Wt and Ifnar1−/− mice were infected with Lm strain LO28InlA*. A, B. Numbers of bacteria in livers (A) and spleens (B) were determined by CFU assay 72 h after intraperitoneal (i.p.) infection with 1×10∧6 Lm. C, D. Bacterial loads of livers (C) and spleens (D) were examined by CFU assay 72 h after intragastric gavage (i.g.) with 5×10∧9 Lm. Plots indicate the Median of bacterial counts. E. 14 mice per group were infected i.g. with 5×10∧9 Lm LO28InlA* and survival was monitored over ten days.

In keeping with previous reports, a drastic difference in the bacterial load was observed in livers and spleens of Wt and Ifnar1−/− mice 72 h after i.p. infection (Figs. 1A, B). When mice were infected i.g., bacterial loads at day three were similar or even slightly elevated in Ifnar1−/− livers and spleens (Figs. 1C, D). To monitor survival we infected Ifnar1−/− and control mice i.g. with a high dose of Lm. IFN-I signalling protected mice from lethal infection with Lm, as Ifnar1−/− mice were the only animals to succumb to infection (Fig. 1E). This is in striking contrast to the results reported after i.p. or intravenous (i.v.) infections [7].

**Figure 2:**
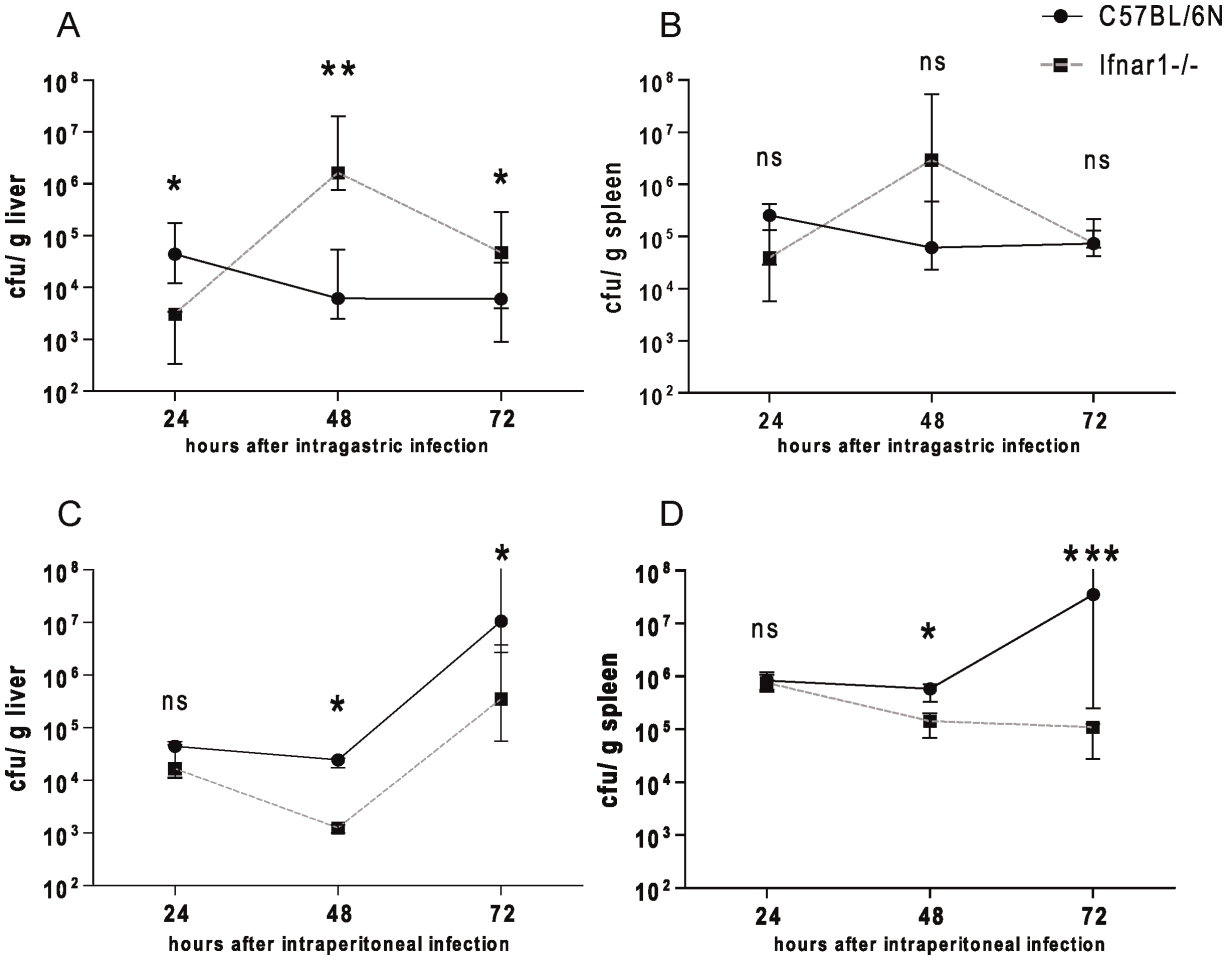
Bacterial burden after various periods of intragastric infections of Wt and Ifnar1−/− mice with *Listeria monocytogenes*. A–B. C57BL/6N Wt and Ifnar1−/− mice were infected with 5×10∧9 CFU of strain LO28InlA* by intragastric gavage (i.g.) and bacterial loads of livers (A) and spleens (B) were monitored over three days by CFU assay. C, D. bacterial burden of livers (C) and spleens (D) was determined by CFU assay over three days after i.p. infection of C57BL/6 Wt and Ifnar1−/− mice with 1×10∧6 CFU of strain LO28InlA*. For i.g. experiments at least 7 mice per genotype and for i.p. experiments at least 5 mice per genotype were used for each time point. Standard variations indicate the median with interquartile range.

The bacterial burden of Lm-infected animals is routinely assessed at day three after inoculation. However, the organ loads shown in figure 1 insufficiently explain the increased mortality of i.g.-infected Ifnar1−/− mice. To determine the time point at which IFN-I exert their protective effects, we monitored bacterial replication between 24 and 72 h after infection (Fig. 2). We detected reduced or equal amounts of bacteria in livers and spleens of Ifnar1−/− mice compared to Wt mice 24 h after infection. 48 h after i.g. infection, Ifnar1−/− mice showed higher numbers of bacteria, with the most striking difference seen in the liver (Figs. 2A, B). In keeping with the results of figure 1 the bacterial loads in Ifnar1−/− mice were only slightly elevated in liver and not at all in spleen compared to Wt levels 72 h after infection (Figs. 1A, B). In striking contrast to these results, i.p. infected Ifnar1−/− mice had lower amounts of Lm in liver and spleen at both 48 h (Figs. 2C, D) and 72 h (Figs. 1C, D) after infection. 24 h after i.p. infection with Lm the organ load was similar in Ifnar1−/− and Wt mice [19].

### The detrimental or beneficial effect of IFN-I is not determined by the size of the L. monocytogenes inoculum

**Figure 3:**
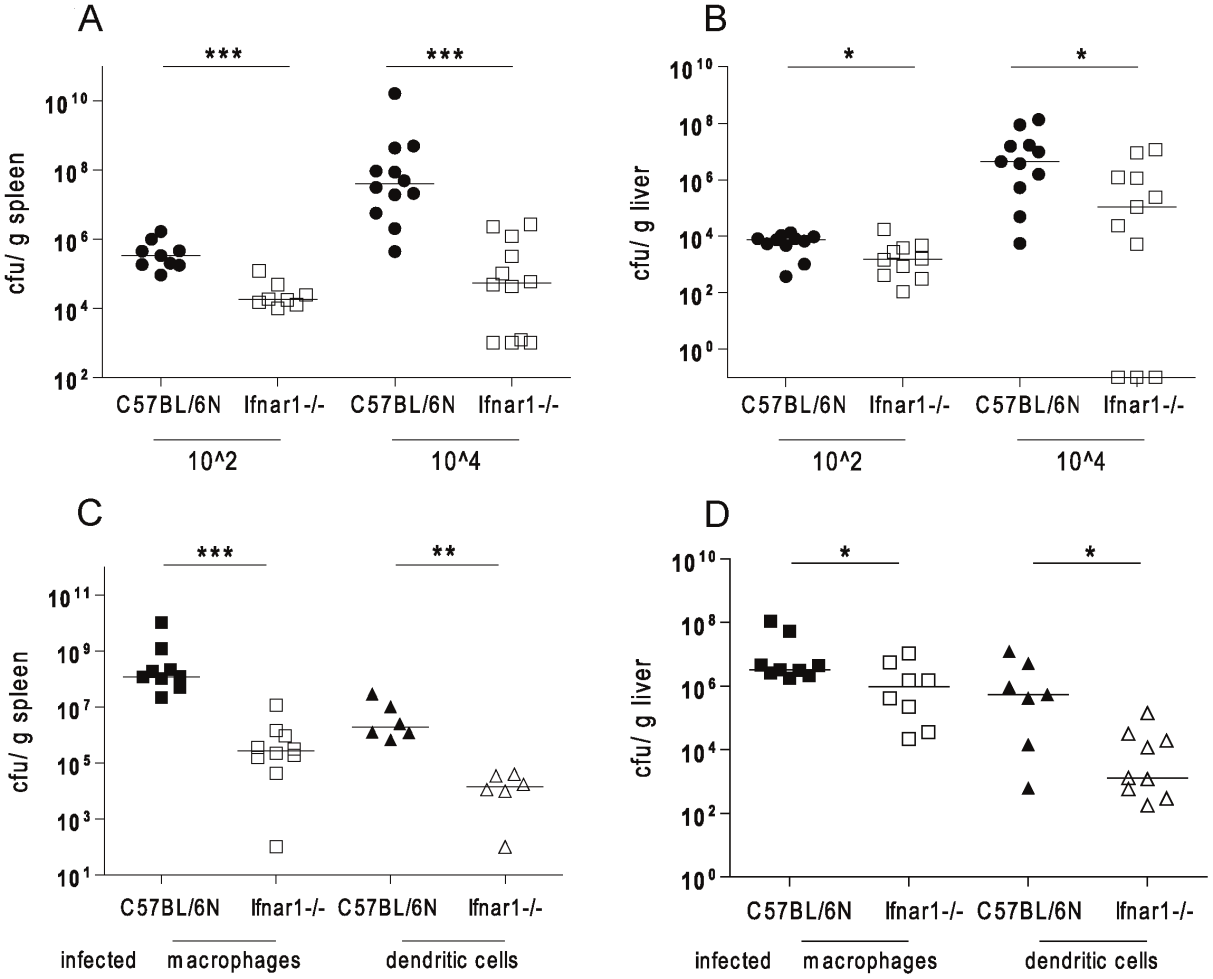
Low infectious doses or dissemination via infected cells do not alter the adverse effect of IFN-I after systemic infection with *Listeria monocytogenes*. A, B,.Doses of10∧2 and 10∧4 Lm were injected intravenously (i.v.) into C57BL/6N Wt and Ifnar1−/− mice and bacterial loads in spleens (A) and livers (B) were determined by CFU assay 72 h after infection. C, D. Wt bone marrow-derived macrophages or myeloid dendritic cells were infected *in vitro* with a MOI of 10 for 1 h, vigorously washed in PBS and 10∧4 cells were injected i.v. into C57BL/6N Wt and Ifnar1−/− mice. The injected populations contained 3–5×10∧3 viable Lm. Bacterial loads in spleens (C) and livers (D) were measured by CFU assay 72 h after infection.

I.p. or i.v. injections of bacteria deliver the entire inoculum directly to internal organs. By contrast, i.g. infection might result in a more gradual release of bacteria from the intestinal tract, hence a lower primary infectious dose for internal organs. To examine whether the amount of the primary inoculum delivered to target organs influences the effect of IFN-I, we infected Ifnar1−/− and control animals with 10^2^ Lm i.v. Figures 3A and B show that at this very low dose Ifnar1−/− mice showed an increased ability to prevent Lm replication, much like mice infected with 100-fold more bacteria. Therefore, parenteral delivery of small inocula does not reproduce the IFN-I effect seen after gastrointestinal administration of Lm. Alternatively, the difference between i.p. and i.g. infection might arise from the mode of dissemination. According to previous reports (e.g. [24]), Lm traversing the intestinal epithelium are taken up and spread throughout the host organism via phagocytic cells residing in mucosal lymphoid organs such as macrophages or DC. We tried to mimic this situation by injecting various amounts of *in vitro* Lm-infected macrophages or dendritic cells i.v. into Wt and Ifnar1−/− mice. 40–50% of these cells harboured bacteria (data not shown). Figures 3C and 3D demonstrate that intracellular delivery of Lm resulted in enhanced bacterial clearance by Ifnar1−/− compared to Wt mice. While these experimental conditions do not faithfully mimic the host-pathogen interactions in the intestinal tract, they show that intracellular dissemination per se does not alter the impact of IFN-I on innate resistance to Lm. The experiment shown in figure 3C and 3D was performed in an identical manner with infected Ifnar1−/− cells and the outcome was highly similar (data not shown). This suggests that under our experimental conditions the response of the infected cells to IFN-I does not contribute to the overall effect of the cytokines on innate resistance to Lm.

### IFN-I do not inhibit invasion of the gut mucosa or of mucosa associated lymphoid tissue

**Figure 4:**
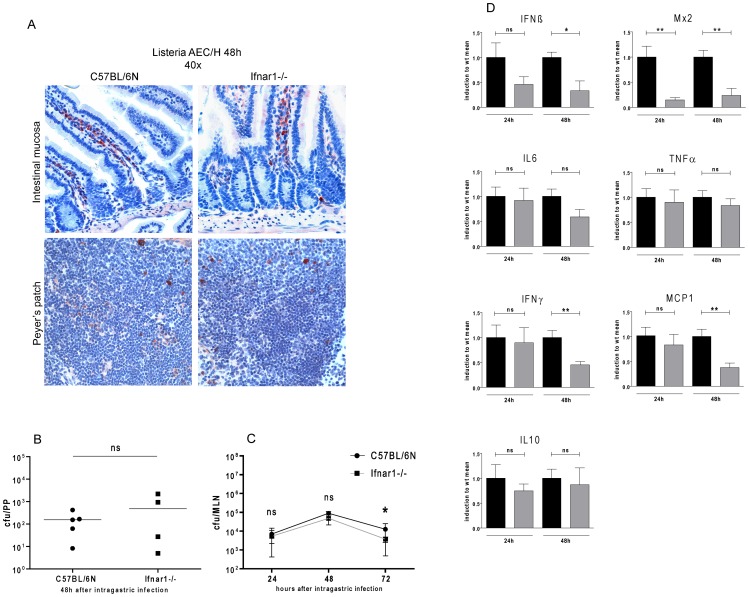
Localization and replication of *Listeria monocytogenes* in the intestinal tract and stimulation of cytokine production in response to infection. A, upper panels. Anti-Listeria serum was used to detect Lm in the intestinal mucosa from C57BL/6N Wt or Ifnar1−/− mice 48 h post infection. Listeria infection, in both Wt and Ifnar1−/− mice occurred mostly in mucosal tissue beneath the epithelial layer. A, lower panels. Anti-Listeria serum was used to detect Lm in Wt or Ifnar1−/− Peyer's patches (PP) 48 h post infection. B, C. Bacterial numbers in PP (B) at day 2 or mesenteric lymph nodes (MLNs, C) over three days, determined by CFU assay. Standard variations for MLN indicate the median with interquartile range from 7 mice (24 h and 48 h time points) or 12 mice (72 h time point). D. Analysis of Peyer's patch mRNAs by qPCR at the indicated times after infection. Mean values and SEM from 9 mice per time point are indicated. All experiments were performed with C57BL/6N Wt and Ifnar1−/− mice infected with a dose of 5×10∧9 CFU of the LO28InlA* strain by intragastric gavage (i.g.).

We tested the hypothesis that the beneficial effect of IFN-I on i.g.-infected mice might result from a decreased rate of intestinal invasion. This is suggested by older reports that epithelial cells treated with IFN-I show increased resistance against invasion by enteropathogens [25], [26]. To monitor the presence of Lm in intestinal tissue or the gut-associated lymphoid tissue we performed immunohistochemistry on the intestinal mucosa 48 h after i.g. infection. Visualization of Lm with a specific antiserum demonstrated the presence of bacteria in mucosal tissue. Very low numbers of Lm were found in epithelial cells, the vast majority had crossed the epithelial barrier to reside in the underlying mucosa (Fig. 4A). No differences between Wt and Ifnar1−/− mice were noted (Fig. 4A, upper panels). Similarly, infection of Peyer's patches (PP; Figs. 4A, lower panel and 4B) or mesenteric lymph nodes (MLN; Fig. 4C) did not reveal an effect of IFN-I up to 48 h after invasion. At 72 h Ifnar1−/− mice showed a slight reduction of CFU in MLN. Together the data reveal little measurable effect of IFN-I on intestinal invasion by Lm. Profiling cytokine mRNA production strengthened the notion that IFN-I have little impact on early events after intestinal invasion. Apart from IFNβ, which is amplified by a positive feedback loop involving IFN-I [3] and the IFN-I inducible Mx gene, there was very little impact of Ifnar1 deletion on cytokine expression in PP (Fig. 4D). 48 h post infection IFNγ and MCP1 production was reduced. At this time the infection is systemic and, as shown below, the control of the immune response most likely dominated by splenic cytokine production.

### Absence of a type I IFN response exacerbates inflammatory pathology in livers of mice infected via the gastrointestinal route

Analysis of the bacterial organ burden during the course of infection demonstrated a pronounced peak of multiplication of Lm in the livers of Ifnar1−/− mice infected i.g. for 48 h. Mice begin to die shortly after this period. Furthermore, our recent demonstration that liver damage is closely correlated to the lethality of infection [22] suggests that IFN-I reduce the severity of liver pathology in i.g.-infected mice. Livers were therefore subjected to histological, immunohistochemical and flow cytometric examination.

**Figure 5:**
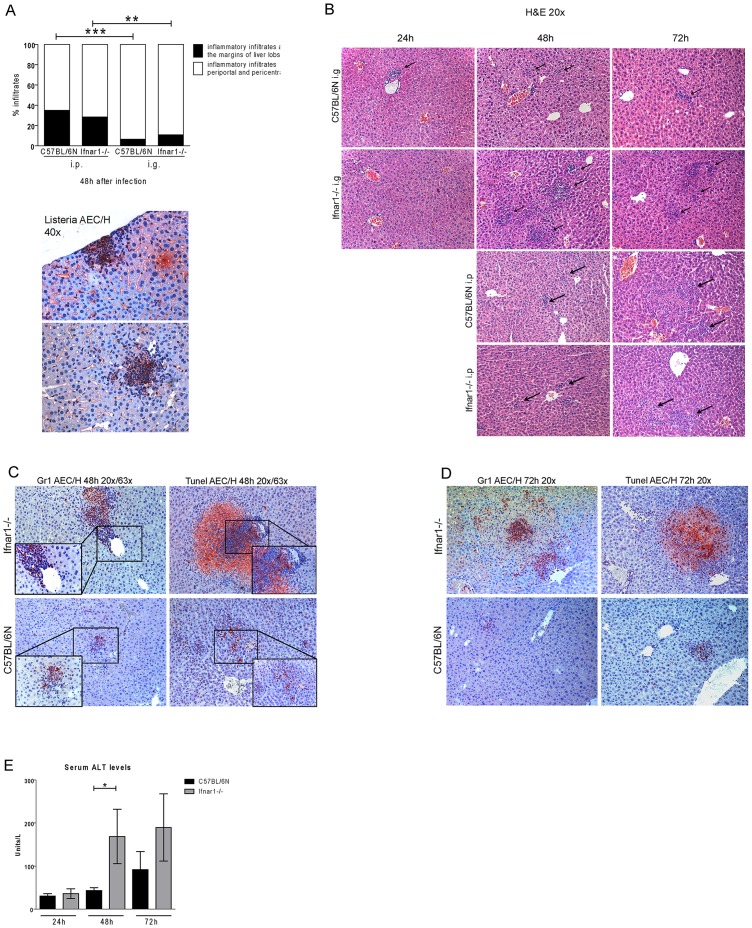
Localization of inflammatory infiltrates in livers of i.p. vs i.g. infected mice. A. Localization of bacteria-containing infiltrates to the margins of the liver lobes (black) or to the periportal or pericentral region (white). The graph indicates relative numbers determined in five C57BL/6N Wt and Ifnar1−/− mice 48 h post i.p. or i.g. infection. The anti-Listeria staining in the panel on the right indicates marginal and periportal infiltrates from a representative Wt sample 48 h post infection. B. Histochemical analysis of hematoxylin-stained liver sections obtained 24 h, 48 h or 72 h after i.g. and 48 h or 72 h after i.p administration of strain LO28InlA* to C57BL/6N or Ifnar1−/− mice. C, D. Gr1+ (left panels) or TUNEL+ cells (right panels) in inflammatory liver infiltrates. Liver sections obtained 48 h (C) or 72 h (D) after i.g administration of strain LO28InlA* to Ifnar1−/− mice were stained with antibody to Gr1 or subjected to TUNEL staining. GR1+ and TUNEL+ cells appear red. E. Serum ALT levels from i.g.-infected mice. The data are representative of two different experiments with seven mice in each group and time point.

To assess whether the route of infection alters the predominant localization of Lm, livers 48 h after i.g. or i.p. infection were subjected to immunohistochemistry with anti-Listeria serum. The vast majority of bacteria colocalized with inflammatory infiltrates (Fig. 5A). Quantitative evaluation revealed a larger fraction of Lm in periportal or pericentral areas after i.g. infection, whereas Lm in i.p.-infected mice showed increased localization at the margins of liver lobes (Fig. 5A).

In accordance with the bacterial loads determined in figure 2, H&E staining of livers after i.g. infection demonstrated an increased number of small inflammatory infiltrates 24 h after infection in Wt compared to Ifnar1−/− livers (Fig. 5B). By contrast, more and much larger infiltrates were observed in Ifnar1−/− mice compared to their Wt controls both 48 h and 72 h after infection. Livers of Ifnar1−/− mice infected i.p. contained very few large-sized inflammatory infiltrates at 48 h. Consistent with bacterial replication (Fig. 2C) the number and size of infiltrates increased in both Wt and Ifnar1−/− livers after 72 h (Fig. 5B). Closer inspection of the inflammatory infiltrate of Ifnar1−/− mice infected for 48 h (Fig. 5C) and 72 h (Fig. 5D) via the gastrointestinal route showed that they contained a large number of Gr1^+^ cells (neutrophils and inflammatory monocytes) and that they were the predominant sites of infection (Figs. 5C, D, right). Strikingly, the infiltrates as well as the surrounding hepatic tissue contained a large number of TUNEL-positive dying or dead cells (Fig. 5 C, D, left), which is consistent with reports that Lm kills infected hepatocytes, macrophages, or dendritic cells [27]. To verify that IFNAR deficiency increases hepatotoxicity after i.g. infection with Lm, serum alanine aminotransferase (ALT) levels were determined. In keeping with our hypotheses, increased serum ALT was found in Ifnar1−/− animals (Fig. 5E).

**Figure 6:**
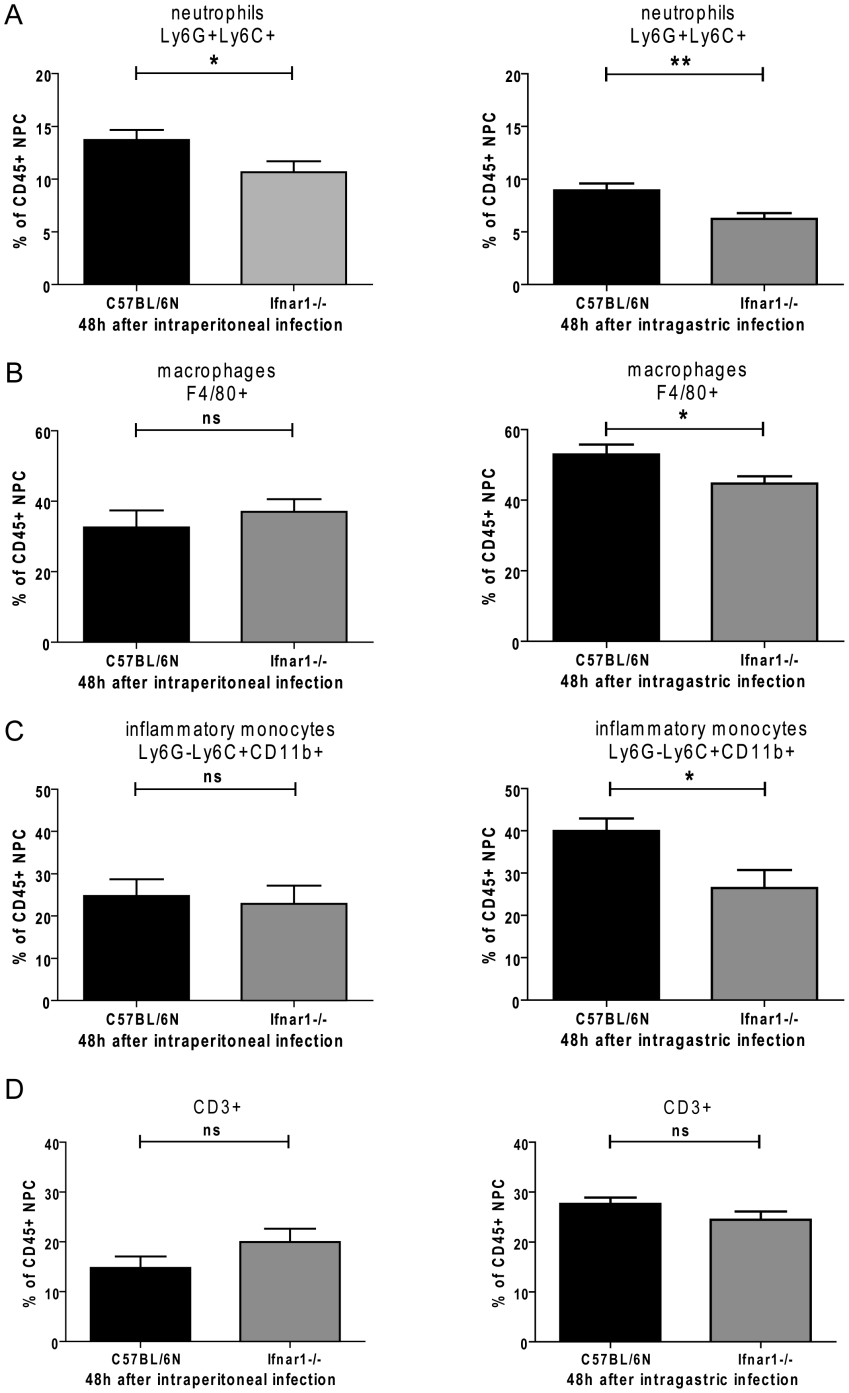
Comparison of the CD45+ fraction of nonparenchymal liver cells (NPC) of i.g.- and i.p.-infected C57BL/6N Wt and Ifnar1−/− mice. A–D. CD45+ nonparenchymal liver cells (NPC) from infected mice were analyzed for neutrophils (A), macrophages (B), inflammatory monocytes (C) and T cells (D) using the indicated markers 48 h post infection. The data are representative of three different experiments with four mice in each group. I.p. infections were performed with doses of 1×10∧6 CFU and i.g. infections with 5×10∧9 CFU of LO28InlA*.

We proceeded to determine the innate effector cell populations present in the fraction of non-parenchymal liver cells (NPC) 48 h after i.p. or i.g. infection. Ifnar1−/− livers contained a somewhat smaller fraction of neutrophils after i.p. injection of Lm and the difference was larger after i.g. infection (Fig. 6A). Ifnar1−/− livers from i.g.-infected mice also contained a slightly smaller proportion of F4/80+ macrophages that include a large fraction of the resident Kupffer cells (Fig. 6B). Notably, i.g. infection of Ifnar1−/− mice caused a slight but significant reduction of the fraction of inflammatory monocytes compared to Wt, whereas this cell type formed an equal percentage of CD45+ cells in both genotypes following infection through the peritoneum (Fig. 6C). The proportion of CD3+ T cells remained largely unaffected by both route of infection and mouse genotype.

**Figure 7:**
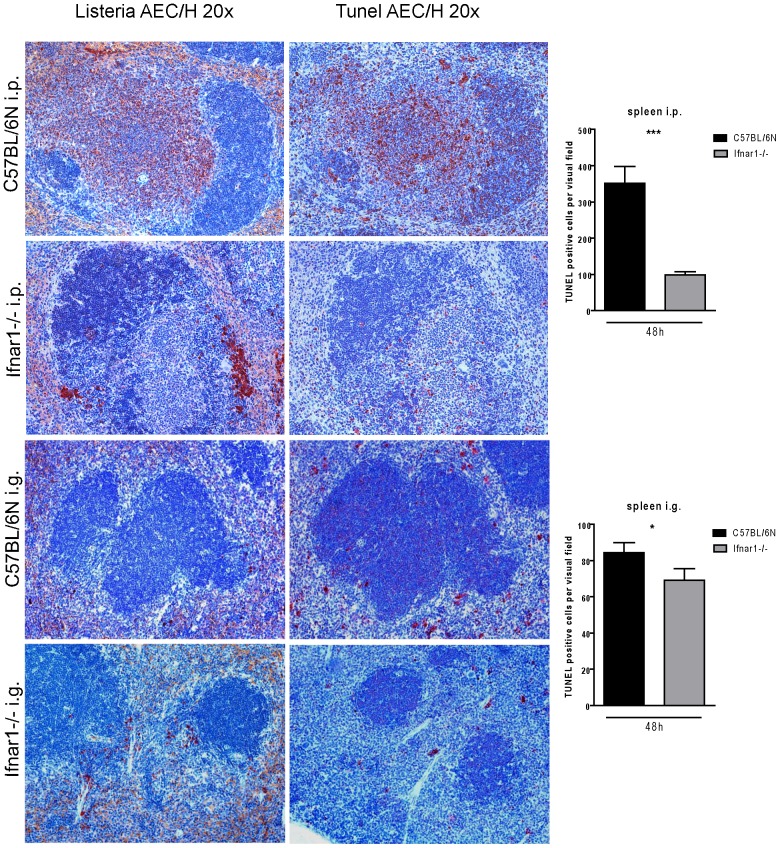
Infection and infection-induced death of splenocytes from i.p.- or i.g.- infected C57BL/6N Wt and Ifnar1−/− mice. Representative samples obtained 48 h after i.p. administration of 1×10∧6 CFU of strain LO28InlA* (upper panels) or i.g. administration of 5×10∧9 LO28InlA* (lower panels) to C57BL/6N Wt or Ifnar1−/− mice as indicated. Panels on the left were stained with anti-Lm antibody. Panels on the right were subjected to in situ TUNEL assay. Infected cells and TUNEL+ cells appear red. The orange background seen in the red pulp of some sections is caused by erythrocyte haemoglobin. The bar graphs show quantification of TUNEL+ cells from sections of four spleens of i.p-infected and six spleens of i.g-infected C57BL/6N (black bar) and Ifnar1−/− (grey bar) mice.

In mice infected through systemic routes, IFN-I strongly enhance the death of splenic T lymphocytes. To assess whether this activity differs after gastrointestinal infection, in situ TUNEL staining of infected splenic tissue was performed 48 h post infection (right panels of Fig. 7) and further compared to staining of serial sections with anti-Lm antibody (left panels of Fig. 7). Consistent with published data [6], [22] the absence of Ifnar1 strongly reduced the number of apoptotic cells in extra-follicular areas of the white pulp after i.p. infection (upper right panels of Fig. 7). Spleens after i.g. infection contained less apoptotic cells in correlation with roughly 10-fold less bacteria (lower right panels of Fig. 7, Fig. 2). In spite of an increase in bacterial numbers relative to Wt controls (lower left panel of Fig. 7, Fig. 2), Ifnar1−/− spleens contained fewer TUNEL+ cells. Thus, the inhibitory activity of IFN-I on splenocyte death appears to be independent of the infection route.

### IFN-I accelerate and increase proinflammatory cytokine activity after intragastric *Listeria monocytogenes* infection

Our analysis of liver inflammation suggests it contributes to, or reflects the different impact of IFN-I on mice infected through enteral or parenteral routes. However, production of cytokines that regulate inflammation and immunity is to a large extent an attribute of leukocytes residing in the blood or in lymphoid organs. Therefore, we determined cytokine mRNA expression in the spleen.

**Figure 8:**
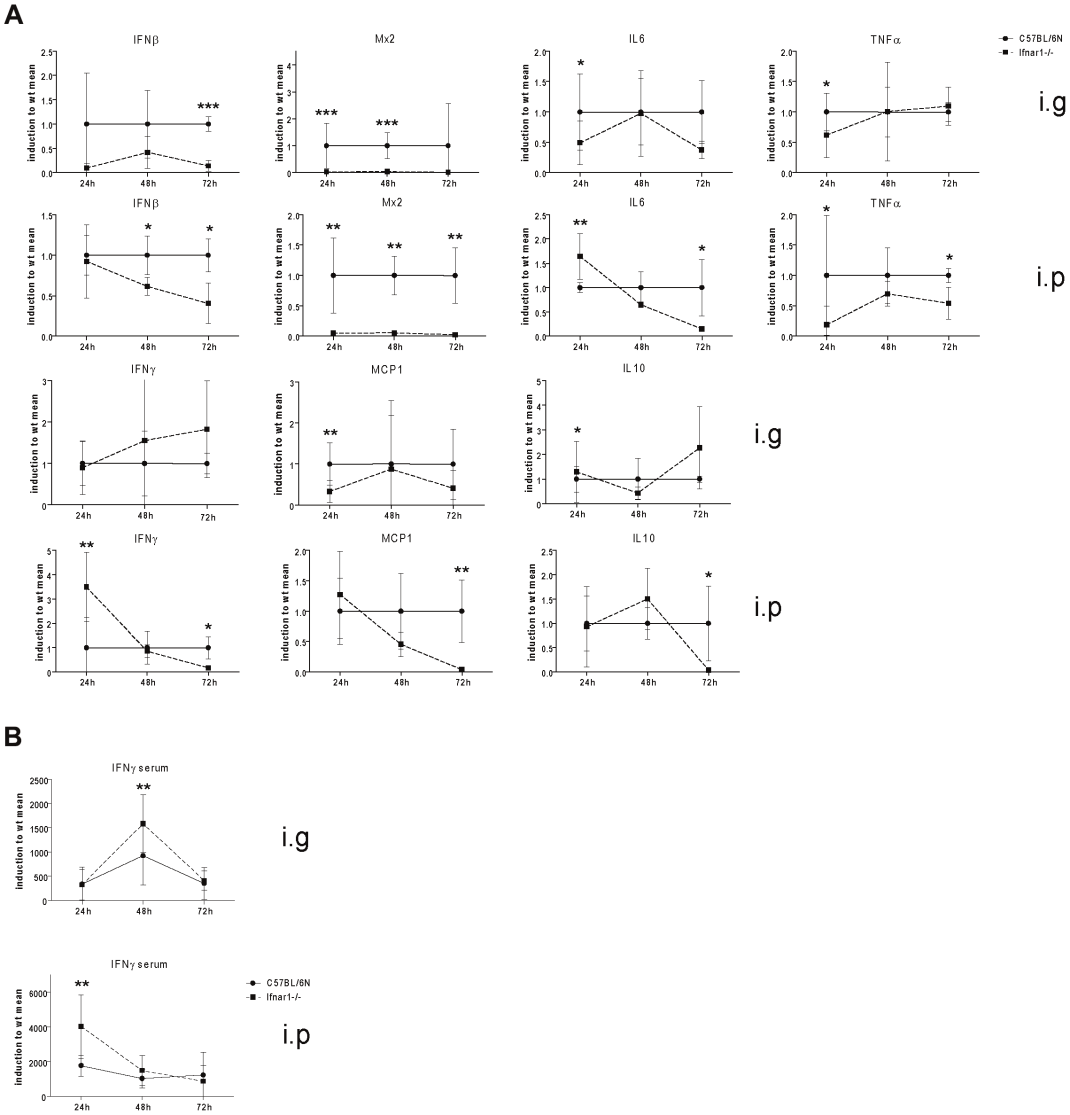
Delayed cytokine response in Ifnar1−/− compared to Wt mice after intragastric infection with *Listeria monocytogenes*. A.Cytokine mRNA expression in the spleens of C57BL/6N (solid line) or Ifnar1−/− mice (hatched line) 24 h, 48 h and 72 h after infection with 5×10∧9 CFU of strain LO28InlA* by intragastric gavage (i.g.), or 1×10∧6 LO28InlA* by intraperitoneal infection (i.p.) was determined by qPCR. mRNAs were normalized to the GAPDH housekeeping control and values obtained from infected Wt mice set to 1. Data from Ifnar1−/− mice thus represent the relative induction compared to the value obtained at the same time point from Wt animals. Mean values and SEM from 9 mice (spleen, all time points of i.g. infection), 7 mice (spleen after 24 h of i.p. infection) or 4 mice (spleen, 48 h and 72 h after i.p. infection) are indicated. B. Serum IFNγ levels of C57BL/6N Wt and Ifnar1−/− mice infected i.g. for 24 h, 48 h or 72 h with 5×10∧9 CFU of strain LO28InlA* by intragastric gavage (i.g.), or with 1×10∧6 CFU LO28InlA* by intraperitoneal injection were determined using a flow cytometry-based bead array. Mean values and SEM from 9 mice (24 h and 48 h time points) or 12 mice (72 h time point) are indicated.

Splenic IFNβ mRNA was expressed up to 72 h after i.g. application of Lm. In accordance with expectations, the IFN-I induced gene Mx2 was expressed in infected spleens up to 72 h in Wt, but was strongly reduced in Ifnar1−/− mice (Fig. 8A). Among the pro-inflammatory cytokines and chemokines tested, IL6 and MCP1 were decreased early after infection. Notably, this effect was not observed at a similar time after i.p. infection. Of further interest, early splenic IFNγ production was increased in Ifnar1−/− mice compared to Wt after i.p. infection, but not after infection through the gastrointestinal tract. Early TNFα production was reduced in Ifnar1−/− mice infected through the intestinal tract and this was more pronounced after i.p. infection. Compared to Wt mice, immunosuppressive IL10 was elevated in Ifnar1−/− 72 h after i.g. infection, but reduced after i.p. infection. The data suggest that splenic cytokine production is an important determinant of the different impact of IFN-I after enteral and parenteral infection routes. Due to the overwhelming importance of IFNγ for innate resistance to Lm [28], [29], delayed production of this cytokine in i.g.-infected versus i.p.-infected Ifnar1−/− mice is likely a major determinant of the difference in innate resistance. It reflects the detrimental increase of hepatic Lm between 24 h and 48 h after i.g. infection. To determine whether systemic IFNγ production corresponds to splenic mRNA, serum IFNγ was measured and found to correlate well with the levels of IFNγ mRNA in the spleen (Fig. 8B).

## Discussion

Lm is a widely studied pathogen. Mouse infection models have been helpful in deciphering innate responses to intracellular bacteria. However, the vast majority of data about immunity to Lm stems from animals infected via the intraperitoneal or intravenous route. Comparably little knowledge has been obtained about infection through the intestinal tract which represents the natural entry route in humans. The incompatibility of the InlA/E-cadherin interaction in murine hosts, required for efficient invasion of epithelial cells, posed an obstacle to murine models of gastrointestinal infection. To overcome this limitation, we followed the approach of Wollert *et al.* [17] to ‘murinize’ the InlA gene for improved interaction with mouse E-cadherin and increased invasion of Lm. The Lm strain LO28 was chosen for this approach because it is a particularly potent inducer of IFN-I *in vitro* and *in vivo* [30]. E-cadherin is located at the basolateral side of epithelial cells, making it less accessible to bacteria in the gut lumen. Pentecost and colleagues have shown that at sites of epithelial cell turnover E-cadherin becomes accessible to Lm [31]. InlB, another Lm invasin, contributes to the initial uptake into host epithelia [32], [33]. Finally, the major virulence factor Listeriolysin O (LLO), a member of the bacterial hemolysin family, promotes the uptake of Lm into hepatocytes [27].

The results of our study clearly show that the route of uptake, hence the initial interactions of Lm with the host, determine the impact of IFN-I on the innate antibacterial response. Strikingly, the impact of IFN-I synthesis changes from being adverse [5], [6], [7] to being beneficial when i.p. and i.g. routes of infection are compared. Both high-dose and low-dose systemic infections produced an adverse effect of IFN-I, suggesting that the switch to beneficial IFN-I action is not caused by a slow release of Lm from the intestine. Likewise, systemic administration of Lm as a passenger of bone marrow-derived macrophage or DC effector cells did not switch the effect of IFN-I. This suggests that dissemination in infected cells does not per se change the impact of IFN. In spite of this we cannot rule out the possibility that dissemination via a cell residing in the gut-associated lymphoid tissue or in the intestinal mucosa would produce a different outcome.

Mechanisms proposed to explain the detrimental actions of IFN-I signalling after i.p. infection are increased T lymphocyte and macrophage death and unresponsiveness of macrophages to IFNγ [34], [35]. Following systemic delivery, IFN-I increase production of immune-suppressive IL10 and restrain TNFα producing cells in the spleen, thereby limiting a protective inflammatory response [5], [9]. Here we show that the production of critical cytokines is altered in absence of type I IFN, either because they are direct targets of IFN-I signalling or because the lack of IFN-I signalling misbalances complex cascades of events that include proinflammatory cytokine synthesis. Among affected cytokines are the protective TNFα, IL6 and IFNγ and the anti-inflammatory IL10, in addition to chemokines such as MCP1/CCL2 that regulate the recruitment of myeloid cells [10], [29], [36], [37], [38]. Unlike early TNFα synthesis that requires IFN-I after both i.p. and i.g. infection, the production of IL6 and MCP1 during the initial 24 h of infection is reduced specifically when bacteria enter their host via the intestine. Most importantly, increased IFNγ production in absence of type I IFN responsiveness was delayed and less pronounced after i.g. compared to i.p. infection. Thus, reduced synthesis of protective cytokines during the early phase of infection provides a likely explanation for the different impact of IFN-I on mice infected via enteral or parenteral routes. This explanation is in accordance with the timing of bacterial replication, which is strongly accelerated in i.g.-infected Ifnar1−/− mice between 24 h and 48 h. The increased IFNγ synthesis at 48 h explains why a significant fraction of Ifnar1−/− mice proceeds to clear bacteria with equal efficiency as their Wt counterparts and survives. We hypothesize that those Ifnar1−/− mice that die from i.g. infection are unable to cope with the damage inflicted by the large Lm burden between 24 h and 48 h. Unexpectedly, intestinal invasion and replication in the intestinal mucosa or the mucosa-associated lymphoid tissue demonstrated little control by IFN-I. In contrast, the liver, most likely the most rapidly infected internal organ, revealed striking differences comparing intragastric or intraperitoneal infection routes, most obviously the vigorous replication of Lm between 24 and 48 h and the correspondingly stronger inflammatory response. Our recent report clearly demonstrated a close correlation between the lethality of Lm infection and the extent of liver damage [22]. It appears likely, therefore, that the hepatic response is a major determinant for the beneficial or adverse effects of IFN-I on Lm infection. Contrasting the spleen, the liver is not itself a site of IFN-I synthesis during Lm infection and it responds poorly to IFN-I [19]. This supports our conclusion that the innate hepatic response is critically influenced by IFN-I-regulated splenic cytokine synthesis. Additionally, tonic signalling by the IFNAR, resulting from low levels of constitutive IFN-I production, plays an important role in priming the innate immune system for appropriately vigorous antimicrobial responses [39]. We cannot exclude the possibility that the lack of such tonic signals in Ifnar1−/− mice contributes to infection-related phenotypes of these animals. However, the lack of tonic IFNAR signalling does provide a straightforward explanation for the different impact of IFN-I after i.g and i.p. infection.

Our data further suggest that in addition to cytokines, the extent of liver inflammation may be influenced by different bacterial entry points after intestinal or intraperitoneal infection routes. Lm arriving from the intestinal tract localizes mostly to periportal or pericentral areas and the inflammatory response is directed to these regions. This suggests that important Lm entry routes are the portal blood stream or, following systemic dissemination, the central veins, with little contribution of peritoneal invasion and direct infection of liver lobes. By contrast, a larger fraction of intraperitoneally administered Lm may choose direct intraperitoneal access to the margins of liver lobules rather than systemic dissemination through blood or lymphatic vessels. Speculatively, the location of replicating Lm may influence the speed with which an inflammatory infiltrate is formed and regulated by blood-borne cytokines and chemokines.

One very obvious cause for liver damage noted in our experiments was the death of cells both within the inflammatory infiltrate and the surrounding hepatic tissue. Cell death may be a direct consequence of intracellular Lm in macrophages, neutrophils or hepatocytes [40], [41], [42] or an indirect consequence of inflammation. Liver infiltrates formed more frequently in i.g.-infected Ifnar1−/− mice and developed to significantly larger sizes, thus causing a much larger fraction of hepatic tissue to die. Livers of Ifnar1-deficient mice contained a smaller fraction of inflammatory monocytes, important antibacterial effector cells [43], specifically following infection via the gastrointestinal route. This may reflect the reduced synthesis of chemokines and contribute to the enhanced multiplication of Lm in the liver.

Apoptosis of splenic lymphocytes is thought to be an important cause for the adverse consequence of IFN-I production after i.p. infection. While spleens from i.g.-infected mice contained less apoptotic cells compared to i.p.-infected controls, absence of the IFNAR1 reduced the number of dying or dead splenocytes despite a higher bacterial burden. This suggests that the enhancement of splenocyte death by IFN-I is established independently of the route of infection.

Taken together our data suggest that the beneficial effect of IFN-I on the innate response to gastrointestinal infection results from ensuring the rapid upregulation of critical protective cytokines that limit hepatic bacterial replication and inflammation. Diminished restriction of bacterial growth in the liver exacerbates hepatic inflammation and damage. Within the IFN-I-regulated cytokine milieu IFNγ, an important determinant of innate immunity, becomes limiting in the early phase of i.g. infection, particularly in absence of other IFN-I-regulated protective proinflammatory cytokines/chemokines. In contrast, i.p.-infected mice benefit from an early boost of IFNγ production in absence of an IFN-I response. Therefore, the influence of the entry route on the kinetics of IFNγ regulation by IFN-I may function as a key determinant of innate resistance. Interestingly, differences in early cytokine production after i.g. infection of Ifnar1−/− mice were more pronounced in spleen than in the gut epithelium or gut-associated lymphoid tissue. This may reflect the fact that splenic leukocytes are a more productive source of IFN-I and generate a larger influence of IFN-I on splenic cytokine/chemokine production. Alternatively, crossing the intestinal barrier may change the expression of Lm genes and alter some important aspect of the interaction with its host. This and other open questions will be addressed in future studies.

## Supporting Information

Figure S1 Comparison of the LO28wt and LO28InlA*. C57BL/6N Wt mice were infected with Lm strains LO28wt or LO28InlA*. Numbers of bacteria in spleens (upper left panel) and livers (upper right panel) were determined by CFU assay 48 h after intraperitoneal (i.p.) infection with 1×10∧6 Lm. Bacterial loads of spleens (lower left panel) and livers (lower right panel) were examined by CFU assay 48 h after intragastric gavage (i.g.) with 5×10∧9 Lm. Plots indicate the Median of bacterial counts.
(TIF)
